# Leveraging trait and QTL covariates to improve genomic prediction of resistance to Fusarium head blight in Central European winter wheat

**DOI:** 10.3389/fpls.2024.1454473

**Published:** 2024-10-04

**Authors:** Laura Morales, Deniz Akdemir, Anne-Laure Girard, Anton Neumayer, Vinay Kumar Reddy Nannuru, Fahimeh Shahinnia, Melanie Stadlmeier, Lorenz Hartl, Josef Holzapfel, Julio Isidro-Sánchez, Hubert Kempf, Morten Lillemo, Franziska Löschenberger, Sebastian Michel, Hermann Buerstmayr

**Affiliations:** ^1^ Department of Forest Genetics and Plant Physiology, Swedish University of Agricultural Sciences, Umeå, Sweden; ^2^ Institute of Biotechnology in Plant Production, Department of Agrobiotechnology, University of Natural Resources and Life Sciences Vienna, Tulln an der Donau, Austria; ^3^ Center for International Blood and Marrow Transplant Research, National Marrow Donor Program/Be The Match, Minneapolis, MN, United States; ^4^ Saatzucht Donau GmbH & CoKG, Reichersberg, Austria; ^5^ Department of Plant Sciences, Norwegian University of Life Sciences, Ås, Norway; ^6^ Institute for Crop Science and Plant Breeding, Bavarian State Research Center for Agriculture, Freising, Germany; ^7^ Summerland Research & Development Centre, Agriculture and Agri-Food Canada/Government of Canada, Summerland, BC, Canada; ^8^ Secobra Saatzucht GmbH, Moosburg an der Isar, Germany; ^9^ Department of Biotechnology and Plant Biology - Centre for Biotechnology and Plant Genomics - Universidad Politécnica de Madrid, Madrid, Spain; ^10^ Saatzucht Donau GmbH & CoKG, Probstdorf, Austria

**Keywords:** wheat, Fusarium head blight, genomic prediction, trait covariates, *Rht-D1*, GWAS, GBLUP

## Abstract

Fusarium head blight (FHB) is a devastating disease of wheat, causing yield losses, reduced grain quality, and mycotoxin contamination. Breeding can mitigate the severity of FHB epidemics, especially with genomics-assisted methods. The mechanisms underlying resistance to FHB in wheat have been extensively studied, including phenological traits and genome-wide markers associated with FHB severity. Here, we aimed to improve genomic prediction for FHB resistance across breeding programs by incorporating FHB-correlated traits and FHB-associated loci as model covariates. We combined phenotypic data on FHB severity, anthesis date, and plant height with genome-wide marker data from five Central European winter wheat breeding programs for genome-wide association studies (GWAS) and genomic prediction. Within all populations, FHB was correlated with anthesis date and/or plant height, and a marker linked to the semi-dwarfing locus *Rht-D1* was detected with GWAS for FHB. Including the *Rht-D1* marker, anthesis date, and/or plant height as covariates in genomic prediction modeling improved prediction accuracy not only within populations but also in cross-population scenarios.

## 1 Introduction

Fusarium head blight (FHB) is a fungal disease of wheat (*Triticum aestivum*) caused by several species of *Fusarium*, including *Fusarium culmorum* and *Fusarium graminearum* (Moreno-Amores et al., 2020b). FHB can lead to yield losses due to reduced grain quality and contamination with mycotoxins, such as deoxynivalenol, which are harmful to human and animal health (Buerstmayr et al., 2020). The inheritance of resistance to FHB is quantitative and complex, controlled by many small-effect loci across the genome. Phenological and morphological traits, such as anthesis date, plant height, and anther retention, have also been implicated as passive mechanisms influencing FHB resistance (Buerstmayr et al., 2020). Notable examples are the reduced height (*Rht*) genes *Rht-B1* and *Rht-D1*, which have been widely introgressed into elite wheat germplasm but have pleiotropic effects on plant height, anther retention, and FHB resistance, where lines with the semi-dwarfing allele have reduced plant height, increased anther retention, and greater susceptibility to FHB (Srinivasachary et al., 2009; Buerstmayr and Buerstmayr, 2016, 2022; He et al., 2016; Akohoue et al., 2022).

Genomics can assist breeding for resistance to FHB. For well-validated, medium- and major-effect quantitative trait loci (QTL), marker-assisted selection (MAS) can be used to introgress favorable alleles from FHB resistance QTL into desirable cultivars (Buerstmayr et al., 2020). For example, the QTL *Fhb1* and *Qfhs.ifa-5A*, which confer type 2 and type 1 resistance, respectively, have been successfully deployed in breeding programs (Buerstmayr and Buerstmayr, 2022). However, the complex inheritance and genotype-by-environment interactions underlying resistance to FHB can reduce heritability estimates and can complicate the identification of associated single nucleotide polymorphisms (SNPs) via genome-wide association studies (GWAS) and QTL mapping (Buerstmayr et al., 2020). In addition, the process of identifying, validating, and employing MAS for one or more FHB resistance QTL is relatively slow and costly (Poland and Rutkoski, 2016). Genomic prediction/selection is a powerful and efficient tool for plant breeding, accelerating the breeding cycle and increasing genetic gain for quantitative traits, such as FHB resistance (Heffner et al., 2010; Heslot et al., 2015; Poland and Rutkoski, 2016; Crossa et al., 2017; Buerstmayr et al., 2020). In contrast to MAS, which considers the effects of individual loci, genomic prediction leverages genome-wide data on SNPs to predict breeding values for the trait(s) of interest (Poland and Rutkoski, 2016; Buerstmayr et al., 2020). One of the most widely used genomic prediction models is genomic best linear unbiased prediction (GBLUP), in which the phenotype is modeled against a relationship matrix estimated from genome-wide SNPs (Meuwissen et al., 2001; Poland and Rutkoski, 2016).

Previous studies have reported that including FHB-correlated traits, which tend to have higher heritability, or FHB-associated SNPs can improve genomic prediction accuracy for FHB resistance (Arruda et al., 2016; Larkin et al., 2020, 2021; Moreno-Amores et al., 2020b, 2020a; Zhang et al., 2021; Akohoue et al., 2022). Several studies have reported results on genomic prediction of FHB resistance within populations, but few have assessed cross-population prediction (Rutkoski et al., 2012; Jiang et al., 2015; Mirdita et al., 2015; Arruda et al., 2016; Herter et al., 2019; Liu et al., 2019; Moreno-Amores et al., 2020b; Verges et al., 2020, 2021; Larkin et al., 2020, 2021; Moreno-Amores et al., 2020a; Sneller et al., 2021; Zhang et al., 2021, 2022; Akohoue et al., 2022; Winn et al., 2023; Garcia-Abadillo et al., 2023).

We sought to leverage the wide existing knowledge on FHB for the improvement of genomic prediction of resistance to FHB across wheat breeding programs. Here, we combined phenotypic (FHB severity, anthesis date, and plant height) and genome-wide SNP data on five Central European winter wheat populations from Austrian and German seed companies, the Bavarian State Research Centre for Agriculture, and the Horizon 2020 SusCrop—ERA-NET “WheatSustain” project for GWAS and genomic prediction. We evaluated whether the inclusion of FHB-correlated traits (i.e., plant height and anthesis date) and/or the *Rht-D1* SNP (identified via GWAS) as covariates in GBLUP improved prediction accuracy for FHB severity in comparison to standard GBLUP in cross-validated (within-population) and cross-population scenarios. To our knowledge, our study is the first to combine both FHB-correlated traits and FHB-associated loci in genomic prediction for FHB. In addition, we describe novel methods for harmonizing data across variable experimental conditions and breeding programs for genomic prediction of FHB within and between populations.

## 2 Materials and methods

### 2.1 Germplasm

We evaluated breeding material from five winter wheat breeding programs in this study: the WheatSustain training set (WTS) and advanced lines (F_6_–F_8_) from the breeding programs of the Bavarian State Research Center for Agriculture (LfL), Saatzucht Donau GmbH & CoKG (Probstdorf/Reichersberg, Austria; SZD), Saatzucht Josef Breun GmbH & CoKG (Herzogenaurach, Germany; BRE), and Secobra Saatzucht GmbH (Feldkirchen, Germany; SEC). The SZD population comprised 2,279 lines. From each of the German breeding programs, advanced lines from two consecutive breeding cycles were evaluated in 2020 and 2021. The 2020 and 2021 breeding cycles from BRE, LfL, and SEC comprised 446 and 543, 148 and 143, and 516 and 269 lines, respectively. Hereafter, the three German breeding programs will be cumulatively referred to as the German population (DEU). WTS comprised 230 winter wheat cultivars and breeding lines, which were chosen with the aim of capturing the genetic variation present in Central and Northern European winter wheat (Isidro y Sánchez and Akdemir, 2021; Garcia-Abadillo et al., 2023).

### 2.2 Phenotyping

The WheatSustain training set was evaluated across seven location-year environments, as described in a previous study (Garcia-Abadillo et al., 2023) (**Supplementary Table S1**). Briefly, WTS was grown in a randomized complete block design with two replications in 2020 and 2021 in Tulln an der Donau, Austria and Vollebekk, Norway and in 2020 in Reichersberg, Austria, and in non-replicated trials in Feldkirchen, Germany in 2020 and 2021. The DEU material was evaluated in non-replicated trials in Feldkirchen, Germany in 2020 and 2021. The SZD population was evaluated in Tulln an der Donau, Austria in a randomized incomplete block design with two replications per year from 2015 to 2022, with an overlap of approximately 30 lines from one year to the next and most lines evaluated in only one year.

In the Feldkirchen trials, all plots were spray inoculated with an *F. culmorum* conidial suspension with a concentration of 1.5 × 10^4^ conidia/mL on 22, 27, and 30 May and on 2 and 8 June in 2020 and on 8, 12, 15, and 22 June in 2021. In the Tulln an der Donau trials, all plots were spray inoculated every other day throughout the entire anthesis period with an *F. culmorum* conidial suspension with a concentration of 2.5 × 10^4^ conidia/mL, and a high level of humidity was maintained with an automated mist irrigation system for 20 h after each inoculation (Buerstmayr et al., 2011; Moreno-Amores et al., 2020a). In the Vollebekk trials, grain spawn inoculum (oat kernels infected with *F. graminearum*) was applied across the field at the booting stage with a density of 10 g/m^2^ followed by daily mist irrigation in the evening until three to four weeks after anthesis (Lu et al., 2013; Tekle et al., 2018). In Reichersberg, grain spawn inoculum (maize kernels infected with *F. graminearum*) was applied across the field four to five weeks before head emergence with a density of 25–30 g/m^2^ (Buerstmayr et al., 2011).

In all trials, the anthesis date (AD) of each plot was recorded when half of the spikes reached anthesis in the plot. In all trials, except for Tulln an der Donau 2016 and Reichersberg 2020, the plant height (PH) of each plot was measured at physiological maturity. In all Tulln an der Donau trials, Fusarium head blight severity (FHB) was scored on a percentage scale (0%–100% infection) at six time points relative to the AD of each plot (approximately 10, 14, 18, 22, 26, and 30 days post-anthesis). In the Feldkirchen 2020 trials, all plots were scored for FHB on 25 and 29 June and on 2 July. In the Feldkirchen 2021 trials, the breeding plots were scored for FHB on 24 June and on 5, 12, and 16 July, while the WTS plots were scored on 24 June and 1, 6, 12, and 18 July. In the Reichersberg 2020 trial, all plots were scored for FHB on 9 July. In the Vollebekk trials, FHB was scored on each plot once between 6 June and 1 July in 2020 and between 12 and 23 July in 2021.

### 2.3 Genotyping

All lines from WTS were genotyped, while a subset from the DEU and SZD breeding programs was selected for genotyping (**Table 1**). Genomic DNA was extracted from one-week-old seedlings and sent for genotyping at TraitGenetics GmbH (Gatersleben, Germany) (Shahinnia et al., 2022; Garcia-Abadillo et al., 2023). Lines from DEU and WTS were genotyped with the 25K Infinium iSelect array (TraitGenetics GmbH, Gatersleben, Germany), while the SZD material was genotyped with the 7K array (TraitGenetics GmbH, Gatersleben, Germany), which is a subset of the 25K array (Shahinnia et al., 2022; Garcia-Abadillo et al., 2023). The physical positions of the SNPs had been previously called against the Chinese Spring reference genome (IWGSC RefSeq v1.0) (IWGSC, 2018; Shahinnia et al., 2022). The SNP data from the 25K array for DEU had been previously filtered for polymorphism, proportion missing < 10%, and minor allele frequency (MAF) > 5% by Shahinnia et al. (2022), resulting in 17,040 SNPs for further analysis. The SZD and WTS data were filtered using the same criteria as DEU, resulting in 6,709 and 19,656 SNPs for further analysis on SZD and WTS, respectively (**Supplementary Table S2**).

**Table 1:** Number of genotypes available for genomic prediction, number of environments in which Fusarium head blight (FHB) severity was evaluated, and genomic heritability for FHB, anthesis date, and plant height within three populations.

Population	N genotypes	N environments	FHB *h^2^_g_*	AD *h^2^_g_*	PH *h^2^_g_*
DEU	1991	2	0.42	0.72	0.39
SZD	643	8	0.50	0.49	0.53
WTS	230	7	0.72	0.99	0.44

FHB, Fusarium head blight; AD, anthesis date; PH, plant height; ***h^2^_g_***, genomic heritability; DEU, German population; SZD, Saatzucht Donau; WTS, WheatSustain training set.

### 2.4 Phenotypic analysis

Because FHB was scored differently across trials, we sought to harmonize the phenotypic data. First, we calculated the number of days between the AD and the date of each FHB observation for each plot (days to score, DTS) due to the sigmoidal relationship between the number of days since initial *Fusarium* infection during anthesis and FHB symptom severity (Garcia-Abadillo et al., 2023). The DTS values were then assigned to 10 time points, approximating the FHB scoring time points used in the Tulln an der Donau trials: (−1) DTS ≤ 0, (0) 0 < DTS ≤ 8, (1) 8 < DTS ≤ 12, (2) 12 < DTS ≤ 16, (3) 16 < DTS ≤ 20, (4) 20 < DTS ≤ 24, (5) 24 < DTS ≤ 28, (6) 28 < DTS ≤ 32, (7) 32 < DTS ≤ 36, and (8) DTS > 36. We then estimated the variance of the FHB observations for each time point within each trial. Garcia-Abadillo et al. (2023) previously analyzed the WTS data described here and found that using FHB data from the time point with the greatest variance in FHB was optimal for genomic prediction across trials. As such, we used FHB data from the time point with the greatest FHB variance within each trial for further analysis. With this strategy, we were able to harmonize the FHB data across trials, with the majority (98%) of the lines present in the full dataset used for further genotype–phenotype analysis (**Table 1**). All AD and PH data were used for further analysis.

For the SZD and WTS populations, which were evaluated in multi-environment trials, we fit the following phenotypic mixed model for each trait with the “breedR” package (Muñoz and Sanchez, 2020) in R (R Core Team, 2020):


$$y_{ijk}=\mu +Gen_{i}+Env_{j}+Rep{\left[Env\right]}_{jk}+GenEnv_{ij}+\epsilon$$

where *y_ijk_* is the FHB, AD, or PH of each plot; *µ* is the overall mean; *Gen_i_* is the fixed effect of genotype *i*; *Env_j_* is the random effect of environment (year for SZD trials, location–year for WTS trials) *j*, *Rep[Env]_jk_* is the random effect of replication *k* nested within environment *j*, *GenEnv_ij_* is the random effect of the interaction between genotype *i* and environment *j*, and *ϵ* is the error. Genotype best linear unbiased estimates (BLUEs) for each trait were extracted for further use in GWAS and genomic prediction.

We calculated trait correlations (*r*) between genotype BLUEs (SZD and WTS) or plot-level values (DEU) for FHB, AD, and PH within each population as:


$$r=\frac{cor\left(trait_{1},trait_{2}\right)}{\sqrt{h_{1}^{2}h_{2}^{2}}}$$

where *cor(trait_1_,trait_2_)* is the Pearson’s correlation between trait 1 and trait 2, and *h^2^_1_* and *h^2^_2_* are the genomic heritability of trait 1 and trait 2, respectively (Sodini et al., 2018). We estimated genomic heritability (*h^2^*) for each trait within each population using the unbiased average semivariance (ASV) method (Feldmann et al., 2022). First, we fit a mixed model for each trait as:


$$y_{i}=\mu +Gen_{i}+\epsilon$$

where *y_i_* is the response vector of genotype BLUEs (SZD and WTS) or plot-level values (DEU) for FHB, AD, or PH; *µ* is the overall mean; *Gen_i_* is the random effect of genotype *i*; and *ϵ* is the error. The variance of the genotype term was modeled as *Kσ^2^_a_*, where *K* is the realized additive genomic relationship matrix and *σ^2^_a_* is the estimated additive genomic variance (Yu et al., 2006; Endelman and Jannink, 2012). We scaled *K* with the ASV method as:


$$K_{ASV}=\frac{K}{\left[{\left(j-1\right)}^{-1}tr\left(K\right)\right]}$$

where *K_ASV_* is the ASV-scaled genomic relationship matrix and *j* is the number of genotypes. We then extracted the genotypic (*σ^2^_g_*) and residual (*σ^2^_ϵ_*) variances to calculate $h^{2}=\frac{\sigma_{g}^{2}}{\sigma_{g}^{2}+\sigma_{\epsilon }^{2}}$ .

### 2.5 Population structure analysis

We used TASSEL version 5.2.85 (Bradbury et al., 2007) to assess population structure across the 3,088 lines from the five breeding programs/populations. We first estimated a distance matrix for the 3,088 lines from the 7K SNP data and then conducted multidimensional scaling (MDS) analysis on the distance matrix. We extracted the eigenvalues of the first five coordinates and the corresponding coordinate values for the 3,088 lines. MDS revealed that the BRE, LfL, and SEC material clustered into one population, hereafter referred to as the German (DEU) population, which was used for further genotype–phenotype analysis.

### 2.6 Genome-wide association analysis

We used TASSEL version 5.2.85 (Bradbury et al., 2007) for GWAS of FHB, AD, and PH within each of the three populations. We fit the following mixed model:


$$y_{i}=\mu +\beta SNP_{i}+Gen_{i}+\epsilon$$

where *y_i_* is the response vector of genotype BLUEs (SZD and WTS) or plot-level values (DEU) for FHB, AD, or PH; *µ* is the overall mean; *SNP_i_* is the fixed effect of each SNP (coded as −1, 0, 1); *β* is the regression coefficient for each SNP; *Gen_i_* is the random effect of genotype *i*; and *ϵ* is the error. The variance of the genotype term was modeled as *Kσ^2^_a_*, where *K* is the realized additive relationship matrix and *σ^2^_a_* is the estimated additive genetic variance (Yu et al., 2006; Endelman and Jannink, 2012). We extracted the SNP p-values and effect estimates from each GWAS model. For multiple test correction of the SNP *p*-values, we conducted a false discovery rate (FDR; α=0.05) analysis for each GWAS model with the “qvalue” package (Storey, 2015) in R (R Core Team, 2020). We used the 25K SNP data for GWAS within DEU and WTS and the 7K SNP data for GWAS within SZD.

### 2.7 Genomic prediction modeling

We used the “remlf90” function in the “breedR” package (Muñoz and Sanchez, 2020) in R (R Core Team, 2020) for all genomic prediction modeling described hereafter. Within each population, we fit the following basic GBLUP mixed model for FHB with five-fold cross-validation with 10 replications:


$$y_{i}=\mu +Gen_{i}+\epsilon$$

where *y_i_* is the response vector of genotype BLUEs (SZD and WTS) or plot-level values (DEU) for FHB, AD, or PH; *µ* is the overall mean; *Gen_i_* is the random effect of genotype *i*; and *ϵ* is the error. The variance of the genotype term was modeled as *K_ASV_σ^2^_a_*, where *K_ASV_* is the ASV-scaled realized additive relationship (kinship) matrix and *σ^2^_a_* is the estimated additive genetic variance (Yu et al., 2006; Endelman and Jannink, 2012; Feldmann et al., 2022). Within each fold of each replication, the values of the validation genotypes were set to missing in the *y_i_* response vector. The kinship matrix was estimated from all genotypes in the population. From each model, we estimated genomic heritability (*h^2^_g_*) as described previously. We extracted the genotype best linear unbiased predictors (BLUPs) from each model as the genomic estimated breeding values (GEBVs) and then calculated prediction accuracy (PA) as:


$$PA=\frac{cor\left(obs,pred\right)}{\sqrt{h_{g}^{2}}}$$

where *cor(obs,pred)* is the Pearson’s correlation between the observed (FHB BLUEs or plot phenotypes) and predicted (GEBVs) values of the validation genotypes and *h^2^_g_* is the genomic heritability.

We also fit the following “trait-assisted” GBLUP mixed model with five-fold cross-validation with 10 replications within each population:


$$y_{i}=\mu +\beta_{c1}C1_{i}+Gen_{i}+\epsilon \text{ and}$$

$$y_{i}=\mu +\beta_{c1}C1_{i}+\beta_{c2}C2_{i}+Gen_{i}+\epsilon$$

where *y_i_* is the response vector of genotype FHB BLUEs (SZD and WTS) or plot-level FHB values (DEU), *µ* is the overall mean, *C1-2_i_* are the BLUEs for AD or PH of genotype *i*, *β_c1–2_* are the regression coefficients for the *C1–2* terms, *Gen_i_* is the random effect of genotype *i*, and *ϵ* is the error. The variance of the genotype term was modeled as *K_ASV_σ^2^_a_*, where *K_ASV_* is the ASV-scaled realized additive relationship (kinship) matrix and *σ^2^_a_* is the estimated additive genetic variance (Yu et al., 2006; Endelman and Jannink, 2012; Feldmann et al., 2022). Within each population, AD and PH were modeled alone and together. Within each fold of each replication, the values of the validation genotypes were set to missing in the *y_i_* response vector. BLUEs (SZD and WTS) or plot-level values (DEU) for AD and/or PH from all genotypes in the population were included in *C1_i_* and/or *C2_i_*. The kinship matrix was estimated from all genotypes in the population. From each trait-assisted model, we extracted the genotype BLUPs and the regression coefficient(s) of the trait covariate(s) and calculated the GEBV of each genotype as


$$GEBV=BLUP+\beta_{c1}C1\text{ or}$$

$$GEBV=BLUP+\beta_{c1}C1+\beta_{c2}C2$$

where *BLUP* is the genotype BLUP, *β_c1–2_* are the regression coefficients for AD or PH, and *C1–2* are the genotype BLUES (SZD and WTS) for AD or PH or plot-level AD or PH values (DEU). We calculated PA as described previously.

A SNP linked to the *Rht-D1* locus was the only SNP significantly associated with FHB in GWAS in all three populations. We tested the use of the *Rht-D1* SNP as a covariate in all GBLUP and trait-assisted GBLUP models previously described, within population with five-fold cross-validation with 10 replications. All terms remain unchanged for each of the previously described models, except for the inclusion of the *Rht-D1* SNP as *β_Rht_Rht_i_*, where *Rht_i_* is the fixed effect of the *Rht-D1* SNP and *β_Rht_* is the corresponding regression coefficient. Within each fold of each replication, the values of the validation genotypes were set to missing in the *y_i_* response vector. SNP values and BLUEs (SZD and WTS) or plot-level values (DEU) for AD and/or PH from all genotypes in the population were included in *Rht_i_* and *C1_i_* and/or *C2_i_*, respectively. The kinship matrix was estimated from all genotypes in the population. From each model, we extracted the genotype BLUPs and the regression coefficients of the trait and *Rht-D1* covariates and calculated the GEBV of each genotype as


$$GEBV=BLUP+\beta_{c1}C1+\beta_{Rht}Rht\text{ or}$$

$$GEBV=BLUP+\beta_{c1}C1+\beta_{c2}C2+\beta_{Rht}Rht$$

where *BLUP* is the genotype BLUP, *β_c1–2_* are the regression coefficients for AD or PH and *C1–2* are the genotype BLUES (SZD and WTS) for AD or PH or plot-level AD or PH values (DEU), *β_Rht_* is the regression coefficient for the *Rht-D1* SNP, and *Rht* is the SNP value (alleles coded as −1, 0, 1). We calculated PA as described previously.

We also tested all previously described GBLUP and trait-assisted GBLUP models with and without *Rht-D1* between all pairs of populations. For each model and for each pair of populations, one population was modeled as the training population and the other as the validation population, and vice versa. For each between-population model, the values of the validation genotypes were set to missing in the *y_i_* response vector. Depending on the model (see previous model descriptions), SNP values and/or BLUEs (SZD and WTS) or plot-level values (DEU) for AD and/or PH from all genotypes from both the training and validation populations were included in *Rht_i_* and/or *C1_i_* and/or *C2_i_*, respectively. The kinship matrix was estimated from all genotypes from both the training and validation populations. We calculated GEBVs and PA as described previously.

## 3 Results

### 3.1 Limited population structure among breeding programs

**Figure 1:**
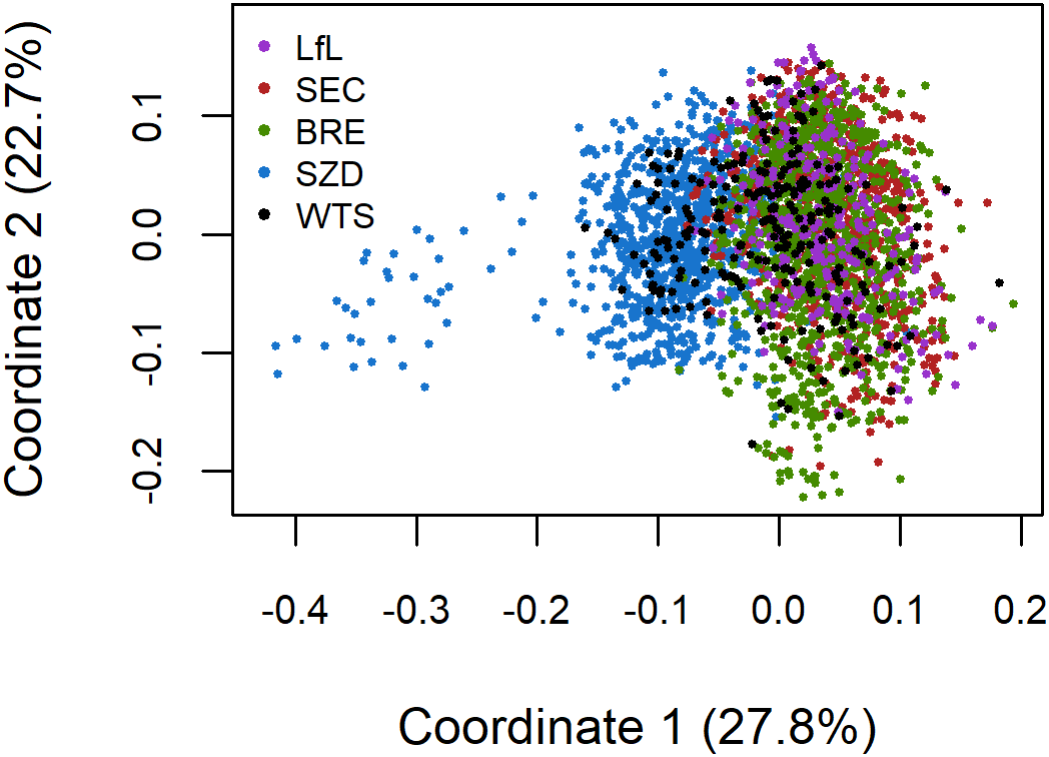
Scatterplot of the first two coordinates from a multidimensional scaling analysis of 3088 lines from the WheatSustain training set (WTS) and the winter wheat breeding programs of Saatzucht Josef Breun (BRE), the Bavarian State Research Center for Agriculture (LfL), Secobra Saatzucht (SEC), and Saatzucht Donau (SZD) using 7K SNP data.

The first and second MDS coordinates explained 28% and 23% of the total variance, respectively. MDS revealed limited population structure, with the first coordinate showing some separation between the Austrian breeding program of Saatzucht Donau (SZD) and the material from the German breeding programs of Saatzucht Breun (BRE), the Bavarian State Research Center for Agriculture (LfL), and Secobra Saatzucht (SEC) (**Figure 1**). BRE, LfL, and SEC were highly genetically related and clustered into one German (DEU) population. The SZD material had greater genetic diversity than the DEU population and the WheatSustain training set (WTS), as demonstrated by the variation among SZD lines on the first coordinate (**Figure 1**). The genetic variation present in WTS overlapped that of the Austrian and German breeding programs (**Figure 1**).

### 3.2 Moderate to high heritability for and correlations between FHB severity, anthesis date, and plant height

Genomic heritability (*h^2^* = 0.42–0.99) was moderate to high for all traits (**Table 1**). WTS had the highest heritability for FHB (*h^2^* = 0.72) and AD (*h^2^* = 0.99), while SZD had the highest heritability for PH (*h^2^* = 0.53) (**Table 1**). Trait correlations were consistent across populations (**Table 2**). FHB was significantly positively correlated with AD (*r* = 0.42–0.64) and negatively correlated with PH (*r* = −[0.22–0.30]) (**Table 2**). AD and PH were significantly positively correlated within DEU and SZD (*r* = 0.38–0.53), but not within WTS (**Table 2**).

**Table 2:** Correlations between Fusarium head blight severity, anthesis date, and plant height within three populations.

Population	Traits	Correlation
DEU	FHB/AD	0.42*
	FHB/PH	−0.22*
	AD/PH	0.38*
SZD	FHB/AD	0.64*
	FHB/PH	−0.27*
	AD/PH	0.53*
WTS	FHB/AD	0.56*
	FHB/PH	−0.30*
	AD/PH	0.03

*p< 0.0001; FHB, Fusarium head blight; AD, anthesis date; PH, plant height; DEU, German population; SZD, Saatzucht Donau; WTS, WheatSustain training set.

### 3.3 *Rht-D1* semi-dwarfing locus associated with FHB severity, anthesis date, and plant height

**Figure 2:**
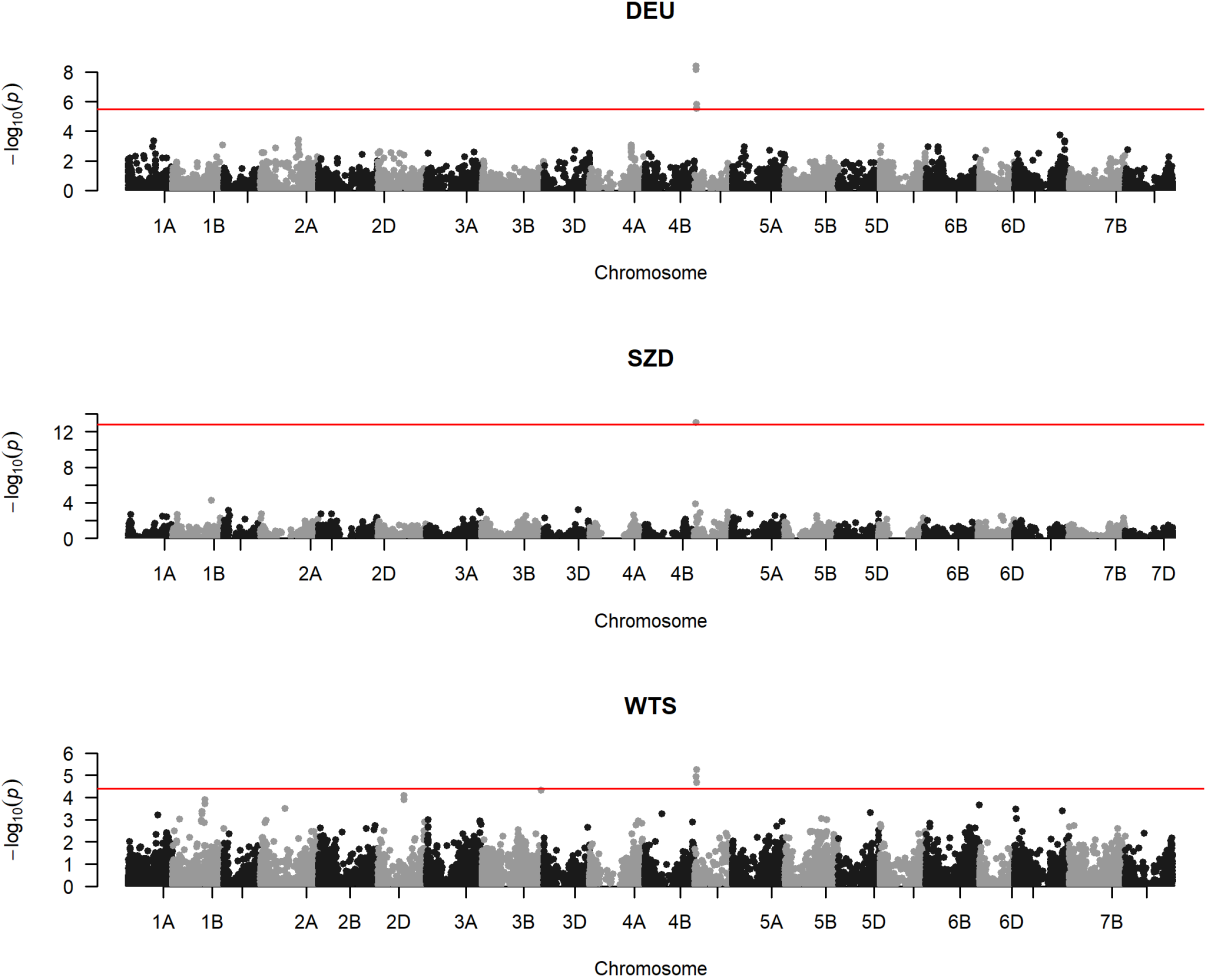
Manhattan plots of genome-wide association studies for Fusarium head blight severity within the German (DEU), Saatzucht Donau (SZD), and WheatSustain training set (WTS) populations, with single nucleotide polymorphism (SNP) physical positions on the x-axis, SNP −log_10_(*p*-values) on the y-axis, and red horizontal lines denoting the false discovery rate threshold for SNP significance.

Within all three populations, one locus (TG0011) was significantly associated with FHB and PH (**Figure 2**; **Table 3**; **Supplementary Figure S1**). Two identical SNPs (TG0011a and TG0011b) underlay this locus within DEU and WTS, which were genotyped with the 25K SNP chip. Only one TG0011 SNP was available for SZD, which was genotyped with the 7K SNP subset. TG0011 is a marker linked to the *Rht-D1* semi-dwarfing gene on chromosome 4D at 18,781,253 bp (Corsi et al., 2021). The wild-type allele reduced FHB by 3%–25% and increased PH by 5–10 cm, relative to the semi-dwarf allele (**Table 3**). Although TG0011 was significantly associated with AD only within WTS, the wild-type allele was associated with reduced AD in all populations (**Table 3**). The *Rht-D1* wild-type and semi-dwarfing alleles were approximately equally represented in the DEU material, while the *Rht-D1* wild-type allele was the major allele within SZD and WTS (**Table 3**).

**Table 3:** Allele frequency and effects on Fusarium head blight (FHB) severity, anthesis date, and plant height of loci significantly associated with FHB within three populations.

Locus	Chr.	Pos. (bp)	Pop.	Allele 1 (freq.)	Allele 2 (freq.)	FHB (%)	AD (days)	PH (cm)
AX-94943274	3B	795,570,750	WTS	G (0.06)	T (0.94)	−5.15^†^	−0.72	4.36
TG0011	4D	18,781,253	DEU	G (0.47)	T (0.53)	−4.5^†^	−0.72	6.49^†^
			SZD	G (0.93)	T (0.07)	−25.09^†^	−1.28	10.33^†^
			WTS	G (0.63)	T (0.37)	−3.11^†^	−1.31^†^	4.55^†^
RAC875_rep_c105718	4D	25,989,047–25,989,414	DEU	C (0.42)	T (0.58)	−3.31^†^	−0.51	3.15^†^
			SZD	C (0.77)	T (0.23)	−6.43	0.08	3.33^†^
			WTS	C (0.54)	T (0.46)	−2.86^†^	−0.30	4.29^†^

^†^Locus significantly associated with trait in GWAS within population (FDR-adjusted *p*< 0.05); GWAS, genome-wide association study; FHB, Fusarium head blight; AD, anthesis date; PH, plant height; DEU, German population; SZD, Saatzucht Donau; WTS, WheatSustain training set; allele effects correspond to the effect of allele 1 relative to allele 2.

Within DEU and WTS, three identical SNPs (RAC875_rep_c105718_672, RAC875_rep_c105718_585, and RAC875_rep_c105718_304) on chromosome 4D at 25,989,047–25,989,414 bp were significantly associated with FHB and PH (**Figure 2**; **Table 3**; **Supplementary Figure S1**). Because SZD was genotyped with fewer SNPs than DEU and WTS, only the RAC875_rep_c105718_672 SNP was available. Within SZD, RAC875_rep_c105718_672 was significantly associated with PH, but not with FHB or AD (**Table 3**). Similar to TG0011, these three SNPs had opposite allele effects on FHB (−[3–6]%) and PH (3–4 cm) (**Table 3**).

Within WTS, one SNP (AX-94943274) on chromosome 3B at 795,570,750 bp was significantly associated with FHB, but not with AD or PH (**Figure 2**; **Table 3**; **Supplementary Figure S1**). AX-94943274 had a moderate effect size on FHB (−6%) and a low MAF (6%) within WTS. This SNP was not included in GWAS within SZD and DEU because it was not on the 7K SNP chip used to genotype SZD and because it had been previously filtered (MAF< 5%) from DEU (Shahinnia et al., 2022).

### 3.4 Anthesis date, plant height, and *Rht-D1* can aid genomic prediction of FHB

Cross-validated (CV) genomic prediction accuracy (PA) for FHB was high (PA = 0.69–0.96) and similar across populations (**Table 4**). Overall, all trait- and *Rht-D1*-assisted models significantly improved PA in comparison to GBLUP, except for GBLUP+PH (**Table 4**). AD-assisted models had lower *h^2^* than all other models overall (**Table 4**). Within all populations, GBLUP+AD, GBLUP+AD+*Rht*, GBLUP+AD+PH, and GBLUP+AD+PH+*Rht* improved PA by 7%–25% compared to standard GBLUP (**Table 4**). Within DEU and SZD, GBLUP models including *Rht-D1* and/or PH without AD did not significantly change PA compared to GBLUP (**Table 4**). Within DEU, GBLUP+AD and GBLUP+AD+PH did not have significantly different PA than their counterparts including *Rht-D1*, suggesting that AD was the key trait for improving PA (**Table 4**). Within SZD, GBLUP+AD+PH and GBLUP+AD+PH+*Rht* had 8–10% higher PA than GBLUP+AD and GBLUP+AD+*Rht*, indicating that modeling both AD and PH as covariates maximized PA (**Table 4**). Within WTS, models including *Rht-D1* had marginally better PA (3%–8%) than their counterparts without *Rht-D1* (**Table 4**). In addition, AD-assisted models, with or without PH, had the best PA within WTS, suggesting that the combination of both AD and *Rht-D1* as covariates can improve prediction accuracy. Genomic heritability was consistent across models within DEU (**Table 4**). Within SZD and WTS, AD-assisted models yielded lower *h^2^* than models not including AD as a covariate (**Table 4**). Within WTS, *Rht-D1*-assisted models had marginally lower *h^2^* than their counterparts without *Rht-D1* (**Table 4**).

**Table 4:** Prediction accuracy and genomic heritability for Fusarium head blight severity of GBLUP models with and without trait and *Rht-D1* covariates within three populations.

Model	DEU		SZD		WTS		Overall	
	PA	*h^2^*	PA	*h^2^*	PA	*h^2^*	PA	*h^2^*
GBLUP	0.73 ± 0.08 B	0.40 ± 0.02 a	0.72 ± 0.10 d	0.51 ± 0.04 a	0.69 ± 0.13 d	0.73 ± 0.06 a	0.71 ± 0.11 e	0.55 ± 0.14 a
GBLUP+*Rht*	0.75 ± 0.07 B	0.40 ± 0.02 a	0.75 ± 0.10 d	0.50 ± 0.04 a	0.76 ± 0.12 cd	0.67 ± 0.06 b	0.75 ± 0.10 d	0.52 ± 0.12 a
GBLUP+AD	0.80 ± 0.07 A	0.40 ± 0.02 a	0.82 ± 0.09 bc	0.47 ± 0.04 b	0.87 ± 0.16 b	0.52 ± 0.09 cd	0.83 ± 0.12 c	0.46 ± 0.08 b
GBLUP+AD+*Rht*	0.82 ± 0.06 A	0.40 ± 0.02 a	0.84 ± 0.09 b	0.47 ± 0.04 b	0.92 ± 0.16 ab	0.49 ± 0.08 d	0.86 ± 0.12 bc	0.45 ± 0.07 b
GBLUP+PH	0.73 ± 0.08 B	0.40 ± 0.02 a	0.75 ± 0.10 d	0.50 ± 0.04 a	0.74 ± 0.10 cd	0.77 ± 0.06 a	0.74 ± 0.09 de	0.56 ± 0.16 a
GBLUP+PH+*Rht*	0.75 ± 0.07 B	0.40 ± 0.02 a	0.77 ± 0.10 cd	0.49 ± 0.04 ab	0.77 ± 0.10 bc	0.73 ± 0.06 a	0.76 ± 0.09 d	0.54 ± 0.15 a
GBLUP+AD+PH	0.82 ± 0.06 A	0.39 ± 0.02 a	0.92 ± 0.10 a	0.42 ± 0.05 c	0.94 ± 0.13 ab	0.54 ± 0.09 c	0.89 ± 0.11 ab	0.45 ± 0.09 b
GBLUP+AD+PH+*Rht*	0.83 ± 0.06 A	0.40 ± 0.02 a	0.92 ± 0.09 a	0.42 ± 0.05 c	0.96 ± 0.14 a	0.52 ± 0.09 cd	0.90 ± 0.12 a	0.45 ± 0.08 b
Overall	0.78 ± 0.08 C	0.40 ± 0.02 c	0.81 ± 0.12 b	0.47 ± 0.05 b	0.83 ± 0.16 a	0.62 ± 0.13 a		

Within each population, each model was five-fold cross-validated (CV) with 10 replications. Means ± standard deviations for PA and *h^2^* are displayed. For comparisons among CV models within each population and across all populations, groups within each table column that are not connected by the same letter are significantly different (Tukey’s HSD test, *p*< 0.05). For comparisons among populations across all models, groups within the last table row that are not connected by the same letter are significantly different (Tukey’s HSD test, *p*< 0.05). DEU, German population; SZD, Saatzucht Donau; WTS, WheatSustain training set; GBLUP, genomic best linear unbiased prediction; AD, anthesis date; PH, plant height; *Rht*, *Rht-D1* single nucleotide polymorphism; PA, prediction accuracy; *h^2^*, genomic heritability. All significance letters should be in lowercase.

Between-population genomic prediction modeling had moderate to high accuracy for FHB (PA = 0.24–0.79) (**Table 5**). Including trait and/or *Rht-D1* as covariates improved PA by 2%–38% over GBLUP for all pairs of populations (**Table 5**). AD-assisted models, with or without PH and/or *Rht-D1*, had the best PA for nearly all pairs of populations; the exception was the scenario where WTS was the training set and SZD was the validation set, for which PH-assisted models had the highest PA (**Table 5**). For three training/validation sets (DEU/WTS, SZD/DEU, and WTS/DEU), *Rht-D1*-assisted models had better PA than their counterparts not including *Rht-D1* (**Table 5**). Under most scenarios, the different models and training/validation sets did not appear to impact *h^2^* (**Table 5**). However, AD-assisted models yielded lower *h^2^* under scenarios where WTS was the training population (**Table 5**).

**Table 5:** Prediction accuracy and genomic heritability for Fusarium head blight severity of GBLUP models with and without trait and *Rht-D1* covariates between three populations.

Model	Training: DEU				Training: SZD				Training: WTS			
	Validation: SZD		Validation: WTS		Validation: DEU		Validation: WTS		Validation: DEU		Validation: SZD	
	PA	*h^2^*	PA	*h^2^*	PA	*h^2^*	PA	*h^2^*	PA	*h^2^*	PA	*h^2^*
GBLUP	0.44	0.36	0.58	0.41	0.13	0.82	0.52	0.70	0.29	0.69	0.50	0.50
GBLUP+*Rht*	0.44	0.37	0.78	0.41	0.17	0.81	0.54	0.69	0.35	0.63	0.44	0.44
GBLUP+AD	0.74	0.35	0.81	0.41	0.30	0.80	0.74	0.67	0.43	0.46	0.31	0.31
GBLUP+AD+*Rht*	0.71	0.36	0.93	0.41	0.32	0.79	0.72	0.69	0.47	0.43	0.28	0.28
GBLUP+PH	0.39	0.37	0.63	0.41	0.16	0.81	0.60	0.60	0.26	0.74	0.57	0.57
GBLUP+PH+*Rht*	0.46	0.37	0.79	0.42	0.18	0.80	0.59	0.68	0.29	0.70	0.52	0.52
GBLUP+AD+PH	0.78	0.35	0.89	0.41	0.33	0.76	0.84	0.63	0.46	0.49	0.33	0.33
GBLUP+AD+PH+*Rht*	0.77	0.35	0.96	0.41	0.34	0.76	0.83	0.63	0.47	0.48	0.32	0.32
Overall (training/val. set)	0.59 ± 0.17 abc	0.36 ± 0.01 c	0.79 ± 0.14 a	0.41 ± 0 c	0.24 ± 0.09 d	0.79 ± 0.02 a	0.67 ± 0.13 ab	0.67 ± 0.03 b	0.38 ± 0.09 cd	0.58 ± 0.12 b	0.54 ± 0.24 bc	0.41 ± 0.11 c

Means ± standard deviations for PA and *h^2^* are displayed. For comparisons of PA or *h^2^* among sets of training and validation sets, groups within the last table row that are not connected by the same letter are significantly different (Tukey’s HSD test, *p*< 0.05). For comparisons among populations across all models, groups within the last table row that are not connected by the same letter are significantly different (Tukey’s HSD test, *p*< 0.05). DEU, German population; SZD, Saatzucht Donau; WTS, WheatSustain training set; GBLUP, genomic best linear unbiased prediction; AD, anthesis date; PH, plant height; Rht, Rht-D1 single nucleotide polymorphism; PA, prediction accuracy; *h^2^*, genomic heritability.

The number of genotypes varied widely among populations, with DEU (N=1991) and WTS (230) having a nearly nine-fold difference in population size (**Table 1**). Within population, population size was negatively correlated with PA (*r* = –0.14; *p*< 0.0001), and the smallest population (WTS) had the best PA and highest *h^2^* overall (**Table 4**). Between populations, the ratio of training versus validation set size was positively correlated with PA (*r* = 0.65; *p*< 0.0001), and the largest population (DEU) had the best PA as the training set but the lowest *h^2^* (**Table 5**). In addition, WTS was the best predicted validation population (PA = 0.67–0.79), while DEU had the worst PA as the validation population (PA = 0.24–0.38) under between-population genomic prediction (**Table 5**).

## 4 Discussion

Here, we evaluated the use of trait and SNP covariates in genomic prediction of FHB resistance within and between three Central European winter wheat breeding populations. Anthesis date and/or plant height were genetically correlated with FHB in all populations. The semi-dwarfing locus *Rht-D1* was associated with FHB and plant height in all three populations and with anthesis date in WTS. The allele effects of the *Rht-D1* SNP on the three traits matched their corresponding phenotypic correlations. The negative correlation between FHB and plant height has been reported many times, while anthesis date has been found to be both negatively and positively correlated with FHB, depending on the population and seasonal weather conditions (Mirdita et al., 2015; Buerstmayr et al., 2020; Moreno-Amores et al., 2020b, 2020a). The *Rht-D1* gene has been previously shown to have pleiotropic effects on plant height and FHB resistance by several studies (Srinivasachary et al., 2009; Buerstmayr and Buerstmayr, 2016, 2022; He et al., 2016; Akohoue et al., 2022).

Cross-validated, within-population PA in our study was comparable to PA for FHB reported in previous studies using GBLUP or ridge regression BLUP, a model similar to GBLUP, in cross-validated scenarios (Rutkoski et al., 2012; Jiang et al., 2015; Mirdita et al., 2015; Arruda et al., 2016; Crossa et al., 2017; Herter et al., 2019; Liu et al., 2019; Moreno-Amores et al., 2020a, 2020b; Verges et al., 2020, 2021; Larkin et al., 2020, 2021; Sneller et al., 2021; Zhang et al., 2021, 2022; Akohoue et al., 2022; Winn et al., 2023; Garcia-Abadillo et al., 2023). We found that trait- and *Rht-D1*-assisted models improved prediction over standard GBLUP in all scenarios. Incorporating FHB-associated SNPs or FHB-correlated traits in genomic prediction modeling has been reported to significantly improve cross-validated PA in some previous studies, but not in others (Rutkoski et al., 2012; Arruda et al., 2016; Herter et al., 2019; Larkin et al., 2020, 2021; Moreno-Amores et al., 2020b, 2020a; Zhang et al., 2022; Garcia-Abadillo et al., 2023).

The SZD and WTS populations had higher overall cross-validated PA and *h^2^* than DEU (**Table 4**). Unlike the German breeding populations, the SZD and WTS populations were evaluated under replicated, multi-environment trials. As such, the estimation of genotypic resistance in SZD and WTS was likely more precise than in the German material, leading to better genomic prediction within population. Although the three populations were not all grown in the same trials and the experimental parameters differed across environments and breeding programs, we believe that our methods were sufficient for harmonizing the phenotypic data for further cross-population analysis. We found similar levels of trait correlations, heritability, and GWAS effect estimates across the three populations, and between-population genomic prediction had moderate to high accuracy. Differences in between-population PA can result from a combination of shared genetic and environmental variance. For example, WTS was grown in the same environments as DEU and SZD, and its population structure overlapped that of the German and Austrian breeding programs, which may have led to its high PA as the validation set under between-population prediction. On the contrary, although the experimental conditions of the DEU population were generally less controlled than those of WTS and SZD, DEU was the best training set under between-population prediction scenarios.

Similar to previous reports on within- and cross-population genomic prediction for FHB, PA tended to be lower between populations than within populations (Hoffstetter et al., 2016; Schulthess et al., 2018; Moreno-Amores et al., 2020a; Verges et al., 2020; Sneller et al., 2021). Cross-population genomic prediction tends to be less accurate than within populations, as genome-wide linkage disequilibrium (LD) structure will differ among populations, ultimately changing associations between traits and markers (Buerstmayr et al., 2020; Isidro y Sánchez and Akdemir, 2021). In general, increased relatedness between the training and validation populations tends to yield higher PA for FHB resistance, which was further supported by our findings (Hoffstetter et al., 2016; Schulthess et al., 2018; Herter et al., 2019; Buerstmayr et al., 2020; Verges et al., 2020). However, genomic relatedness alone did not capture all shared variation between populations, as demonstrated by the improvement of GBLUP including anthesis date and/or the *Rht-D1* SNP as covariates in most between-population scenarios. Trait correlations shared among populations are not subject to the same dynamics as LD structure, and our results indicate that FHB-correlated traits can complement genomic prediction for FHB, which may be the result of linkage between or pleiotropy at QTL for FHB resistance and FHB-correlated traits (Schulthess et al., 2018; Steiner et al., 2019; Moreno-Amores et al., 2020b, 2020a; Verges et al., 2020; Sneller et al., 2021; Zhang et al., 2022). Here, the allele effects of the *Rht-D1* SNP on FHB and FHB-correlated traits were shared among populations and including *Rht-D1* SNP as a covariate improved PA, suggesting that linkage phase at this locus was similar across populations (Herter et al., 2019).

Here, we sought to minimize the confounding effects of maturity on FHB severity by timing *Fusarium* inoculations and FHB scoring based on anthesis. However, AD was correlated with FHB, and AD-assisted models improved genomic prediction between and within populations, suggesting that our experimental procedures did not fully control the phenological relationship between anthesis timing and FHB symptom development. Previous studies found that modeling phenology and environmental patterns improved genomic prediction for FHB (Moreno-Amores et al., 2020a; Garcia-Abadillo et al., 2023), further demonstrating the importance of phenotyping phenological traits such as AD in FHB trials.

To our knowledge, our study is the first to (a) model both FHB-correlated traits and *Rht-D1* together as covariates in genomic prediction for FHB resistance in a (b) harmonized dataset of diverse FHB trials from both public and private breeding programs. Although the phenotypic data generated by the partners of the WheatSustain consortium differed with respect to the number/timing of FHB observations and trial environmental parameters (location, year, and inoculation method), our methods allowed not only for the harmonization of the dataset across trials but also for moderate to high PA for FHB between breeding programs. For collaborations between breeding companies and/or public institutions, we recommend that cross-population genomic prediction for FHB resistance be aided by the agronomically important and easily measurable traits of plant height and anthesis date and by markers for semi-dwarfing genes.

## Data Availability

The datasets presented in this study can be found in online repositories. The names of the repository/repositories and accession number(s) can be found in the article/**Supplementary Material**.

## Supplementary material

Supplementary Figure 1 Quantile-quantile plots of the expected vs. the observed SNP *p*-values from GWAS of FHB severity within the German (DEU), Saatzucht Donau (SZD) and WheatSustain training set (WTS) populations.

Supplementary Figure 2 Linkage disequilibrium plot of chromosome 4D in the German population, with *R^2^* values in the upper diagonal and *p*-values in the lower diagonal. The region flanked by the significant SNPs from GWAS of FHB severity is outlined in black.

Supplementary Figure 3 Linkage disequilibrium plot of chromosome 4D in the Saatzucht Donau population, with *R^2^* values in the upper diagonal and *p*-values in the lower diagonal. The region flanked by the significant SNPs from GWAS of FHB severity is outlined in black.

Supplementary Figure 4 Linkage disequilibrium plot of chromosome 4D in the WheatSustain training set, with *R^2^* values in the upper diagonal and *p*-values in the lower diagonal. The region flanked by the significant SNPs from GWAS of FHB severity is outlined in black.
