# Supplementary figures and images for: Leveraging trait and QTL covariates to improve genomic prediction of resistance to Fusarium head blight in Central European winter wheat
Source: Front Plant Sci. 2024 Oct 4;15:1454473. doi: 10.3389/fpls.2024.1454473 (PMC11486744; doi:10.3389/fpls.2024.1454473)

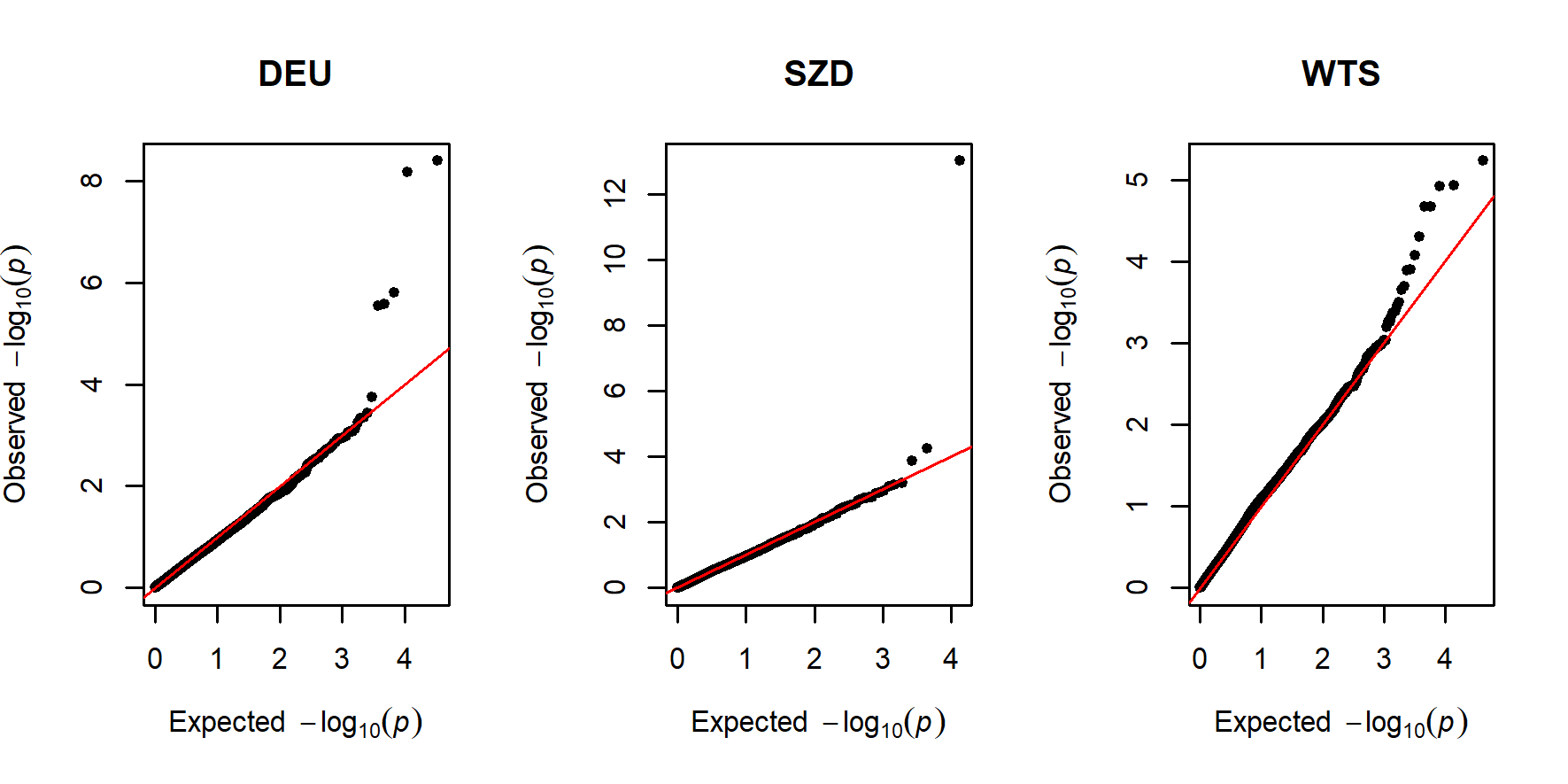

Supplement: Supplementary Figure 1 — Quantile-quantile plots of the expected vs. the observed SNP p-values from GWAS of FHB severity within the German (DEU), Saatzucht Donau (SZD) and WheatSustain training set (WTS) populations. [file Image1.tif]

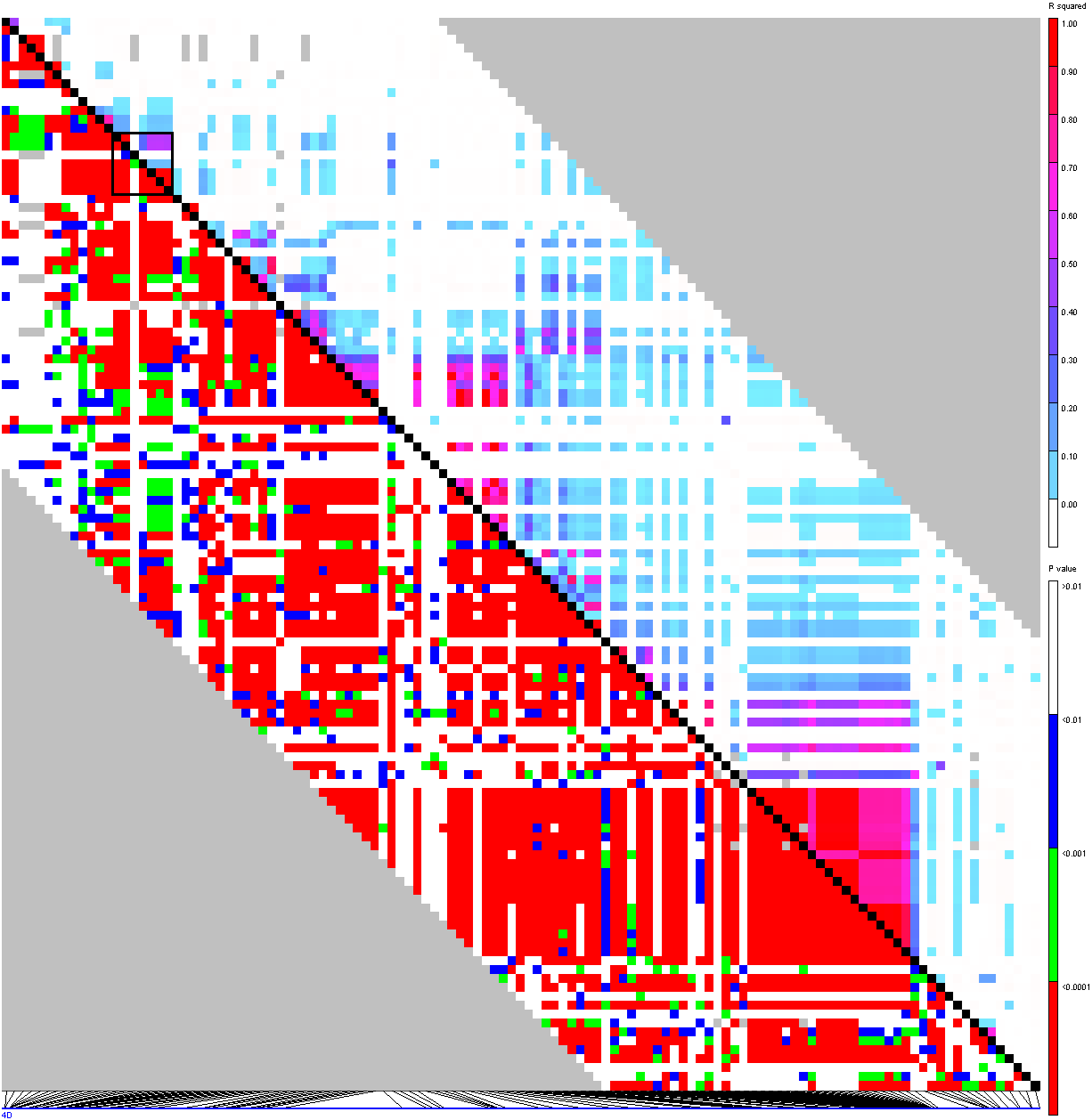

Supplement: Supplementary Figure 2 — Linkage disequilibrium plot of chromosome 4D in the German population, with R2 values in the upper diagonal and p-values in the lower diagonal. The region flanked by the significant SNPs from GWAS of FHB severity is outlined in black. [file Image2.tif]

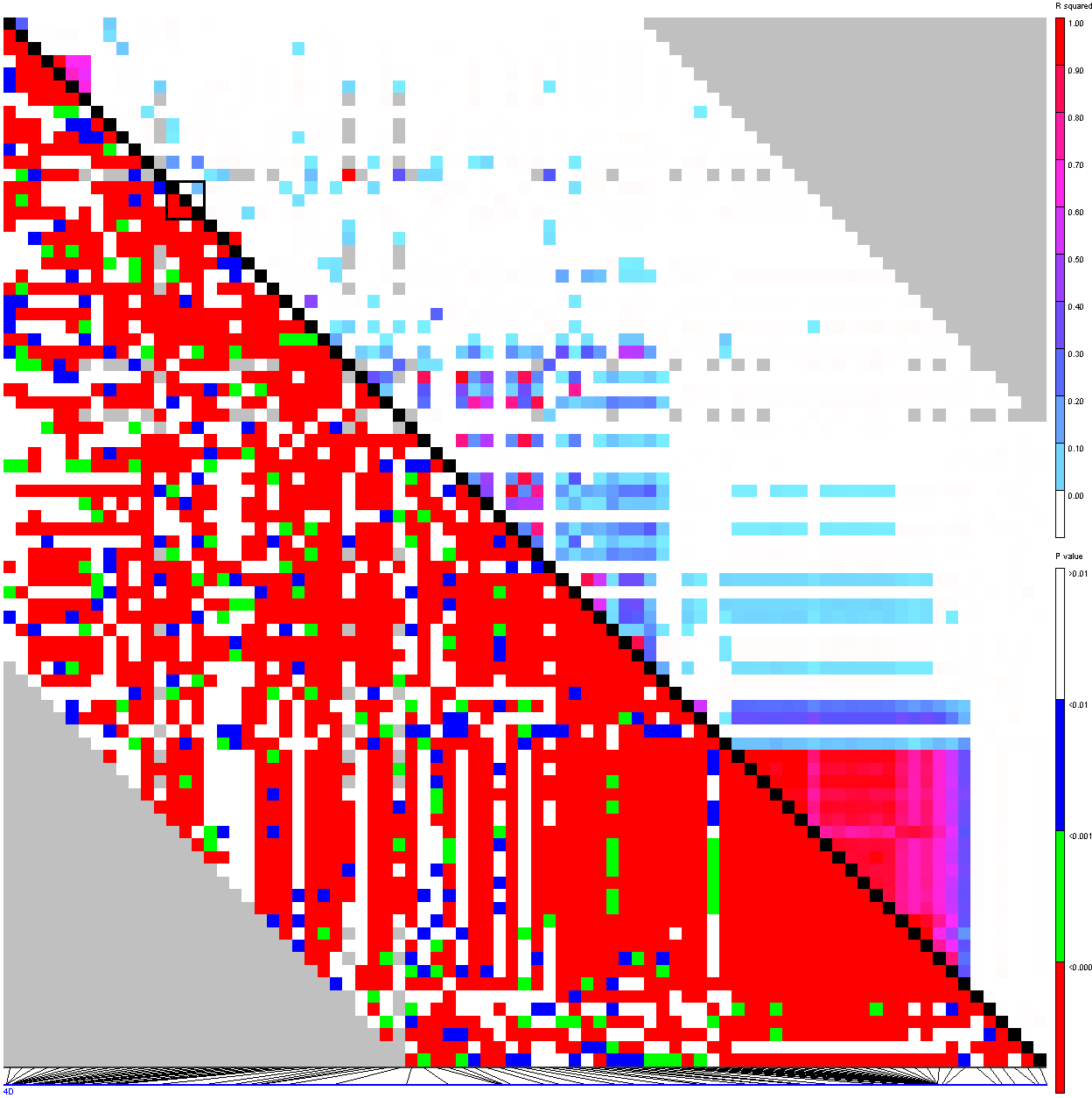

Supplement: Supplementary Figure 3 — Linkage disequilibrium plot of chromosome 4D in the Saatzucht Donau population, with R2 values in the upper diagonal and p-values in the lower diagonal. The region flanked by the significant SNPs from GWAS of FHB severity is outlined in black. [file Image3.tif]

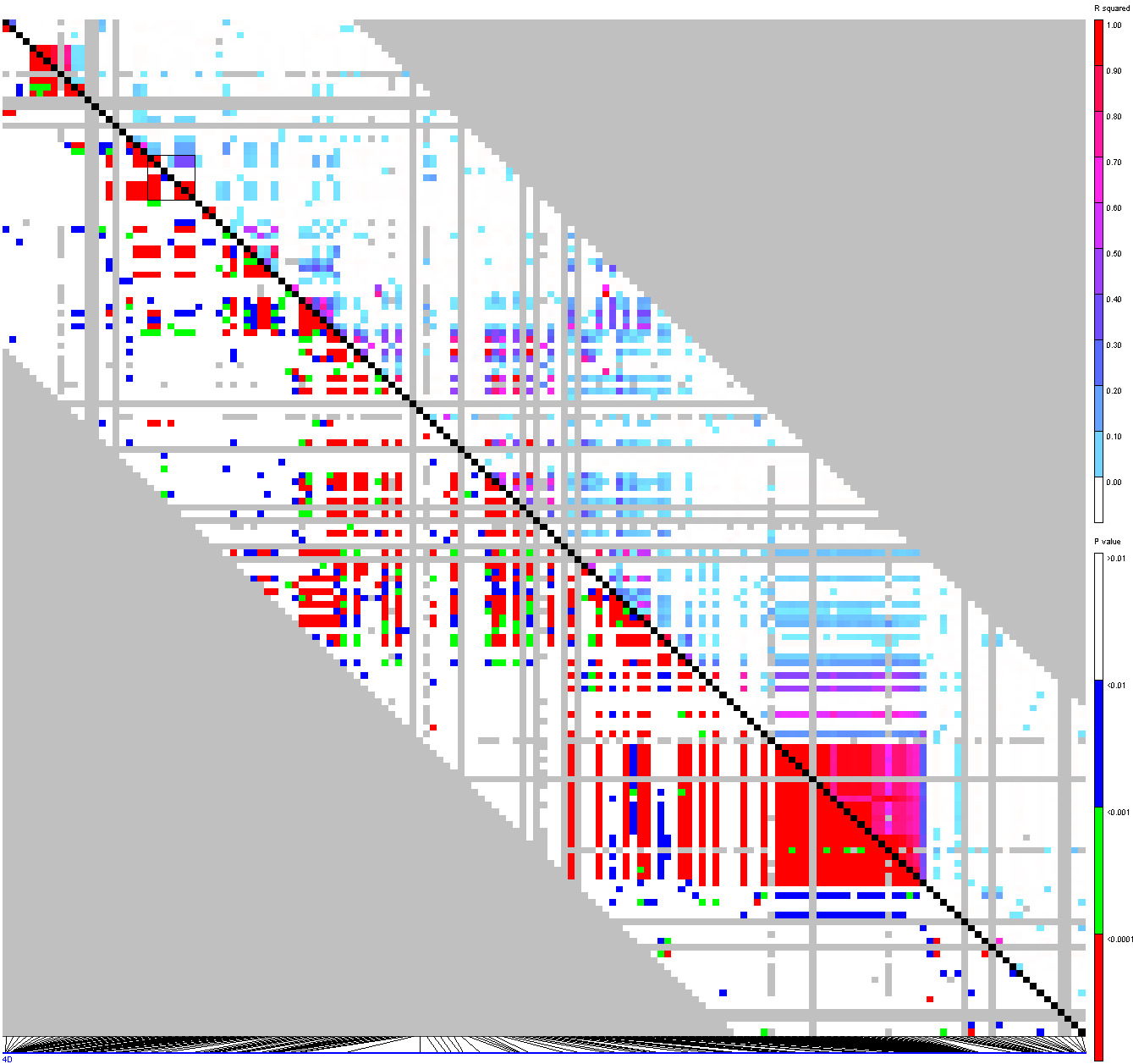

Supplement: Supplementary Figure 4 — Linkage disequilibrium plot of chromosome 4D in the WheatSustain training set, with R2 values in the upper diagonal and p-values in the lower diagonal. The region flanked by the significant SNPs from GWAS of FHB severity is outlined in black. [file Image4.tif]
